# A Nationwide Population-Based Study of Corrosive Ingestion in Taiwan: Incidence, Gender Differences, and Mortality

**DOI:** 10.1155/2016/7905425

**Published:** 2015-12-27

**Authors:** Chuan-Mei Chen, Yueh-Chin Chung, Li-Hung Tsai, Yi-Chen Tung, Horng-Mo Lee, Mei-Ling Lin, Hsin-Li Liu, Woung-Ru Tang

**Affiliations:** ^1^School of Nursing, College of Medicine, Chang Gung University, No. 259, Wenhua 1st Road, Guishan District, Taoyuan City 33302, Taiwan; ^2^Department of Nursing, Central Taiwan University of Science and Technology, Taichung, Taiwan; ^3^Department of Nursing, Chang Gung University of Science and Technology, Taoyuan, Taiwan; ^4^School of Medical Laboratory Sciences and Biotechnology, Taipei Medical University, Taipei, Taiwan

## Abstract

Corrosive injury results from the intake of corrosive-acid-based chemicals. However, this phenomenon is limited to a small number of cases and cannot be extrapolated to the epidemiology of corrosive injuries in actual situations. This study focuses on the annual incidence of corrosive injury and its connection to gender, risk factors, and in-hospital mortality. All patients with corrosive injury (ICD-9 947.0–947.3) were identified using a nationwide inpatient sample from 1996 until 2010. Chi-squared tests and multivariate logistic regression were used to examine risk factors of gender differences and in-hospital mortality of corrosive injury. Young adults comprised the majority of patients (71.2%), and mean age was 44.6 ± 20.9 years. Women showed a higher incidence rate of corrosive injuries, age, suicide, psychiatric disorder, and systemic complications compared with men (*p* < 0.001). The present study demonstrated that age (OR = 10.93; 95% CI 5.37–22.27), systemic complications (OR = 5.43; 95% CI 4.61–6.41), malignant neoplasms (OR = 2.23; 95% CI 1.37–3.62), gastrointestinal complications (OR = 2.02; 95% CI 1.63–2.51), chronic disease (OR = 1.30; 95% CI 1.08–1.56), and suicide (OR = 1.23; 95% CI 1.05–1.44) were strongly associated with in-hospital mortality. Educational programs may be helpful for reducing the incidence of ingestion of corrosive chemicals.

## 1. Introduction

Corrosive agents are broadly divided into acids, alkalis, organophosphates, and other harmful chemicals. The clinical features of corrosive ingestion include airway edema and stomach perforation in acute stages. Dependent upon the quantity and type of corrosive agents used and the duration of exposure, in the chronic stages, corrosive ingestion may also result in lifelong esophageal stricture and an increase in the risk of cancers and other diseases [1–3]. Estimations indicate that US health care organizations have spent a total of 22.9 million US dollars or 28,860 US dollars per patient for corrosive injuries in the USA [4, 5].

Based on the 2013 Annual Report of the American Association of Poison Control Centers, 175,594 subjects suffered ingestion of corrosive chemicals. Ingestion of corrosive chemicals in children ranks second among all causes of poisoning, while deliberate ingestion of corrosive substances in adults ranks third [6]. The occurrence of corrosive ingestion in children is mostly accidental, but the majority of adult cases (70.5%) are intentional self-harm [2, 6]. Psychiatric disorders and socioeconomic stresses are considered the most common reasons for corrosive ingestion [7]. Legislative efforts to identify individuals with psychosocial distress for appropriate therapy are important for reducing the incidence of deliberate ingestion of corrosive chemicals. Indeed, the incidence of corrosive injuries has significantly declined due to legislative efforts and more rigorous packaging [5]. Unfortunately, the occurrence rate of corrosive ingestion remains high in most developing countries. Thus, identifying subjects with suicidal tendencies and psychiatric disorders is important from a preventive viewpoint. However, most epidemiological studies use data collected from the retrospective analysis of cases where patients ingested corrosive agents in a relatively limited time frame. The limited number of participants may not reflect the actual situation in large cohorts [5, 8]. Corrosive injuries in children were reported using Kids' Inpatient National Database [9] or the National Electronic Injury Surveillance System Database [5], but no nationwide population database has been used to investigate factors that contribute to the causes and outcomes of corrosive injuries. The aims of the present study are (1) to estimate the annual incidence and sex-specific incidence of corrosive injury and (2) to examine the risk factors associated with the occurrence of in-hospital deaths caused by corrosive injury in Taiwan.

## 2. Materials and Methods

### 2.1. Data Source

The Taiwan National Health Insurance (NHI) program was launched in 1995 and covers most of the population. The National Health Insurance Research Database (NHIRD) was established in 1997 and collects all claims of those insured in the NHI program. In 2013, more than 23.4 million individuals enrolled in the NHI (coverage rate > 99% of the population) [10]. The data source in the present study was the NHIRD, which was made available through the National Health Informatics Project (NHIP) in Taiwan. NHIP provides scientists with datasets for research purposes. The database comprises comprehensive information on insured persons, including demographic data, diagnostic codes, dates of clinical visits and hospitalization, length of hospital stay, details of prescriptions, procedures/surgeries, and expenditures. NHIP is one of the largest nationwide, population-based datasets in the world. More than 300 studies based on the NHIRD have been published in peer-reviewed journals.

### 2.2. Study Sample

Data from subjects, who were admitted to hospitals between January 1, 1996, and December 31, 2010, were collected from the NHIRD. The inclusion criteria for discharge diagnosis (primary diagnosis) of corrosive injury were established on the basis of the International Classification of Diseases, Ninth Revision Clinical Modification (ICD-9-CM): 947.0–947.3. Patients tagged with ICD-9-CM: 947.4–947.9 (noningestion corrosive agent patients) were excluded. A total of 16,001 subjects with an initial primary diagnosis of corrosive injury were admitted.

### 2.3. Variable of Interest

The following variables were obtained from the NHIRD: patient age, sex, hospital characteristics, socioeconomic status, suicide, in-hospital mortality, length of stay, comorbidities, and complications.

Comorbidities, complications, and suicide were defined according to relevant ICD-9-CM codes. Comorbidity in epilepsy is defined as other conditions may precede, cooccur with, or follow the diagnosis of corrosive injury. The comorbidities assessed included cerebrovascular disease (codes 430–438), diabetes (code 250), lower respiratory disease (codes 491–494, 496), liver cirrhosis (code 571), chronic renal failure (codes 581–586, 403.91–403.93), psychiatric disorder (codes 290–316), and the top five malignant neoplasms, namely, malignant neoplasm of trachea, bronchus, and lung (code 162); malignant neoplasm of liver (code 155); primary malignant neoplasm of hepatic flexure colon (codes 153-154); malignant neoplasm of female breast (code 174); and malignant neoplasm of upper lip, tongue, gum, mouth, palate, tonsil, hypopharynx, lip, oral cavity, and pharynx (codes 140–146, 148, and 149). Patients diagnosed with psychiatric disorder were classified into depressive disorders (296.2x–296.3x, 300.4, 311.x), generalized anxiety disorders (293.84, 300.0x, 300.10, 300.2x, 300.3, 300.5, 300.89, 300.9, 308.x, 309.81), combined depression and anxiety, and other psychiatric disorders [11, 12].

The complications of corrosive injury were also classified into systemic and gastrointestinal (GI) complications. Systemic complications include aspiration pneumonia (code 5070), respiratory failure (codes 51881, 51882, and 51883), DIC (code 2866), septicemia (codes 038.0–038.9), and acute renal failure (codes 584, 5849). GI complications consist of GI bleeding (codes 53082, 5310, 53100, 53101, 5320, 53200, and 53201), GI perforation (codes 5304, 5311, 53110, 53111, 5321, 53210, and 53211), GI bleeding and perforation (codes 5312, 53120, 53121, 5322, 53220, and 53221), corrosive esophagitis (codes 53010, 53019), peritonitis (codes 567, 567.9), fistula (codes 53089, 53084, and 5374), and stricture and stenosis of esophagus (code 5303). The ICD-9-CM code for suicide was E950–E959.

### 2.4. Statistical Analysis

The annual incidence and sex-specific incidence of corrosive injury for 1996–2010 were examined. Annual incidence rates were calculated by dividing the weighted estimate for all annual admissions for corrosive injury in Taiwanese hospitals by the annual civilian population of Taiwan using estimates from census data [13].

Demographic data were described using mean and standard deviations for normally distributed continuous variables, frequencies, and percentages for categorical variables. Bivariate analyses were performed using chi-squared tests to examine the association between each study variable and gender in corrosive injury patients. Multivariate logistic regression was used to obtain the OR and 95% CI to analyze the concurrent effects of various factors on the occurrence of in-hospital deaths. All the statistical tests were two sided with a significance level of *p* < 0.05. Analyses were performed using SPSS version 18.

## 3. Results

The total number of corrosive injury cases in Taiwan between 1996 and 2010 was 16,001 (8,991 female patients, 7,010 male patients). Patient age ranged from 0 to 114 years, with the mean age of 44.6 (±20.9) years. A total of 1,243 subjects (7.8%) were under the age of 18 years (Table 1) and 796 subjects (4.9%) were under the age of 10 years. The above results suggest that the ingestion of corrosive agents is not prevalent in children and in a young generation.

**Figure 1:**
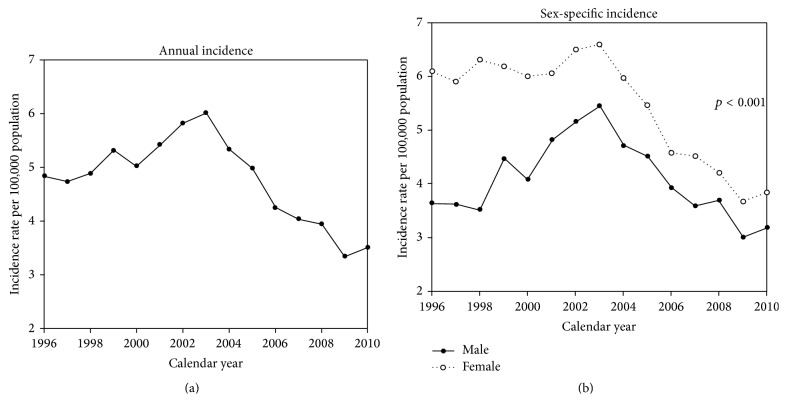
Incidence of corrosive injury between 1996 and 2010. (a) Annual incidence; (b) sex-specific incidence.

Between 1999 and 2003, the annual incidence rate of corrosive injuries was 5 per 100,000 population; however, this incidence rate decreased thereafter. From 2008 to 2010, the average incidence rate was less than 4 per 100,000 population. The incidence rate of corrosive injury was significantly higher in females than in males (*p* < 0.001) (Figure 1).

Suicide and a history of psychiatric disorders were the most common reasons for ingesting corrosive substances. A total of 6,134 subjects (38.3%) had the intention to commit suicide (Table 1) and 3,687 subjects (23.0%) had a history of psychiatric disorders. Among psychiatric disorders, depression was the most common comorbidity. Complications caused by ingesting corrosive substances were investigated. During hospitalization, 11.5% of subjects reported systemic complications, and of these, 6.0% were respiratory failure and 3.9% were aspiration pneumonia. Alternatively, 8.5% of subjects reported GI complications, mainly corrosive esophagitis (3.8%) (Table 2).

Among 6,134 subjects with the intention to commit suicide, a significantly higher incidence was found in women (3,888 subjects) than in men (2,246 subjects) (*p* < 0.001). Women also reported more histories of psychiatric disorders (*p* < 0.001) and systemic complications (*p* = 0.001). Conversely, women exhibited shorter lengths of hospital stay compared with men (8.97 ± 11.12 versus 9.40 ± 11.60, *p* < 0.05) (Table 3).

Results of the multivariate analysis showed that systemic complications (OR = 5.43; 95% CI 4.61–6.41) and GI complications (OR = 2.02; 95% CI 1.63–2.51) were strongly associated with in-hospital mortality caused by corrosive injuries. For comorbidities, malignant neoplasms (OR = 2.23; 95% CI 1.37–3.62) and chronic disease (OR = 1.30; 95% CI 1.08–1.56) also contributed to in-hospital mortality. Moreover, suicide (OR = 1.23; 95% CI 1.05–1.44) was shown to be a risk factor for in-hospital mortality. Lastly, based on subjects aged 0 to 18 years, which served as the reference group, results showed that mortality increased with age, especially for people above the age of 65 years (OR = 10.93; 95% CI 5.37–22.27) (Table 4).

## 4. Discussion

Compared with previous studies that were limited to small samples of ingested corrosive agents, the present study analyzed all incidences with the whole Taiwanese population as the denominator, an approach that substantially reduced bias. To date, no previous studies have used nationwide insurance health data to investigate corrosive injuries. This study is the first to focus on gender differences, and results showed that female patients have a relatively high proportion of suicide intention, psychiatric disorder, and systemic complications.

In Western countries, the incidence of corrosive esophageal injuries declined due to legislative efforts and stricter packaging standards [5, 8]. Similarly, incidence in Taiwan has started to decrease since 2004. Unfortunately, the same situation may not be the case in developing nations [14]. Mortality rates caused by caustic ingestion are reported to be as high as 20% [15], indicating that individual lives are still threatened by acute and chronic complications or mortality due to corrosive injuries. Such situation is one of the most challenging concerns in health clinical practice; thus, prevention is of utmost importance.

In America, nearly 50% to 80% of corrosive injury incidences occur in children [3, 9, 16, 17]. In Taiwan, however, only 8% of incidences were reported on persons aged 18 years and below. Research conducted by Ananthakrishnan et al. [18] shows comparable results with those observed in Taiwan. In India, the incidence rate among children is approximately 15%. Another research conducted in Nigeria indicates that the incidence in children is 25% [19]. The results illustrate that the incidence of children with corrosive esophageal injury is lower in Asian and African countries compared with that in America. No research showing the difference and possible reasons is available to date. To confirm whether such results vary across countries, further investigation is necessary.

The mean age for the incidence of corrosive injuries is 44.6 ± 20 years, and 71.2% of subjects were young adults. Current findings are consistent with findings obtained in other countries [19–21]. Typically, young adults that constitute this group are in a period of life when they are most creative and productive in terms of overall working capacities. The said incidences inhibit young adults from engaging in work forces, which subsequently causes socioeconomic problems. Furthermore, health expenses related to the treatment of corrosive injuries present a heavy burden on NHI institutions [22].

Results of the present study indicate that females are more likely to suffer corrosive injuries than males. Between 1996 and 2010, the incidence rate of corrosive injuries was 3.3–6.0 per 100,000 population in females compared to 3.0–5.5 per 100,000 population in males. Such findings are consistent with previous studies [20–23]. Consequently, female subjects show higher intentions of suicide than men. According to Satar et al. [21], most victims were housewives, who do more cleaning and washing compared with men. Housewives often use sodium hypochlorite as a household bleach in cleaning toilet bowls, as opposed to other cleaning agents. The said agents are not expensive and are easily accessible; however, containers for the agents often do not include warnings. Thus, women can easily use such agents to commit suicide, which may explain why the ingestion of caustic agents occurred more in women than in men. Therefore, increased emotional support is needed for women with suicidal intention to reduce the incidence of corrosive injuries.

Numerous studies have reported that patients who ingest caustic substances exhibit accompanying psychiatric disorders, including depression, anxiety, schizophrenia, adjustment problems, and personality disorders [19, 24, 25]. Results of the present study showed that 23.0% of subjects exhibited psychiatric disorders. This finding is consistent with the results of Adedeji et al. [19]. Women suffer higher levels of depression and generalized anxiety compared with men but have lower levels of difficulties in the category of other psychiatric disorders (*p* < 0.001). As such, medical professionals could allocate resources more effectively to females with depressive and generalized anxiety disorders and to males with other psychiatric disorders.

Patients with corrosive injuries were also observed to suffer from systemic complications. Respiratory failure accounted for 6.0% of the complications, while aspiration pneumonia accounted for 3.9%. Similarly, Tseng et al. [26] showed that 4% of corrosive injury patients suffered from aspiration pneumonia, though another Taiwanese study showed aspiration pneumonia (11.36%) and respiratory failure (7.69%) at much higher levels [27] compared to the findings of the present study. Nonetheless, because Chang et al. only used data from medical centers [27], as opposed to the Taiwanese national medical database, the current study shows a more comprehensive profile of corrosive injury incidences across a greater spectrum of medical care facilities.

The GI complications of GI bleeding (1.5%) were also observed to be similar to the findings by Chang et al. [27]. However, the incidence of stricture and stenosis of the esophagus accounted for 1.3% in the present study, an incidence rate that is lower than that reported in previous studies. The incidence of esophageal strictures after corrosive ingestion has been reported to be between 5% and 73% [28, 29], but the majority of previous studies indicate incidence rates of 10%–19.5% [24, 30–32]. The noticeable difference in percentage levels between the two studies can be attributed to factors such as the nature and concentration of the corrosive substances, intake volume of victims, and time of exposure [33, 34]. However, the present study reports esophageal stricture and stenosis at considerably lower incidence level (1.3%), which is possibly connected to the long-term complications and symptoms that may arise within three months or one year after [2] the original event. The present study only utilizes the first hospitalization data to perform analysis, and the average length of stay is 9.16 ± 11.33 days. By contrast, other studies utilized chart reviews to track the status of patients with chronic complications. Therefore, the present study reports a lower rate of esophageal stricture and stenosis. The above findings provide researchers with an understanding of the experience or conditions of patients with esophageal stricture and stenosis. Health workers should use precautionary or preventive measures for patients to preclude esophageal strictures during short lengths of hospitalization. Nonetheless, esophageal stricture may lead to reflux esophagitis, which may be an aggravating factor that causes refractory stricture of the esophagus; hence, regular observation and aggressive antiacid therapy are necessary for patients with corrosive esophagitis [35].

The present study also showed that systemic complications (OR = 5.43; 95% CI 4.61–6.41) and GI complications (OR = 2.02; 95% CI 1.63–2.51) were strongly associated with in-hospital mortality. In-hospital mortality was observed to be significantly higher in subjects above the age of 65 years compared with subjects aged between 0 and 18 years (OR = 10.93; 95% CI 5.37–22.27). Elderly subjects with systemic complications (i.e., respiratory failure, aspiration pneumonia, septicemia, acute renal failure, and DIC) due to caustic ingestion were observed to exhibit the highest in-hospital mortality (23.3% versus 7.3%, *p* < 0.001) (data not shown). This finding is consistent with the results of Chang et al. [27]. Therefore, strict management, including supportive care with maintenance of nutrition and sepsis control, is crucial for preventing complications in the initial stage.

In addition, approximately 38.3% of subjects with corrosive injuries exhibited suicidal intentions, a finding similar to the results of Ogunleye et al. [36]. The subjects with suicidal intentions were also significantly higher for in-hospital mortality compared to the subjects with no suicidal intentions (OR = 1.23; 95% CI 1.05–1.44). In the same way, a significant relationship among suicidal intentions and malignant neoplasms (*p* = 0.008), chronic disease (*p* = 0.013), and psychiatric disorder (*p* < 0.001) (data not shown) was also observed. The case control study by Cheng [37] reports that the risk for suicidal intentions was significantly associated with psychiatric conditions in Taiwan. Other related studies report that a history of hospitalization for psychiatric disorders is related to the highest odds ratio of intent to commit suicide [38, 39]. Druss and Pincus [40] showed an association between pulmonary diseases and increase in chances within a lifetime of suicidal conceptualization. Likewise, the likelihood of a suicide attempt increases when patients are afflicted with cancer. Björkenstam et al. mentioned similar ideas that indicate links between medical conditions and suicidal intentions, wherein greater levels of severity in illnesses resulted in significantly higher risks of suicide [41]. Thus, healthcare workers need to pay increased attention to patients with psychiatric disorders, malignant neoplasms, and chronic diseases.

## 5. Conclusion

Results of the analyses performed in this study showed marked variations in female patients with a high incidence rate of corrosive injuries. Age, suicide, psychiatric disorder, and systemic complications were significantly higher in females than in males. Furthermore, patients with age, systemic complication, malignant neoplasms, GI complications, chronic disease, and suicidal tendencies exhibited higher in-hospital mortality rates. Healthcare workers could disseminate educational information on psychiatric care targeted for the female population, including providing a secure environment and ensuring more rigorous packaging and warnings for this particular group. For patients who are expected to develop systemic complications, clinicians need to develop preventive treatment plans. Finally, future studies are needed to gather information on how and why it occurs and develop additional preventive strategies.

## 6. Limitation

This study has a number of limitations that should be taken into account when interpreting the findings. First, the extent and scope of tissue damage are related to the types of corrosive agents, the format of the substance, the corrosive agent concentration, and the amount of intake. However, the National Health Insurance Research Database (NHIRD) cannot provide the abovementioned information. As a result, the relationship between systemic complications and GI complications could not be explored. Second, corrosive injury patients with private insurance cannot necessarily recognize their suicidal intention when seeking medical treatments due to the reinvestment from insurance criteria. Hence, the ratio of suicidal intention may have been underestimated in light of the actual situation. Lastly, reliability and validity of the secondary data in the NHIRD could not be verified, although the NHI Bureau features a medical review system to avoid fake or tentative diagnoses.

## Figures and Tables

**Table 1:** Demographic data of corrosive injury patients, 1996–2010, *N* = 16,001.

Variables	*N* (%); mean ± SD^a^; range
Age	44.6 ± 20.9
0–18	1243 (7.8)
19–39	5918 (37.0)
40–64	5476 (34.2)
Above 65	3364 (21.0)
Sex	
Male	7010 (43.8)
Female	8991 (56.2)
Hospital characteristics	
Medical center	14,128 (88.3)
Regional hospital	1768 (11.0)
Local hospital	103 (0.6)
Clinics	2 (0.1)
Socioeconomic status	
Low-income households	271 (1.7)
Non-low-income households	15730 (98.3)
Suicide	6134 (38.3)
In-hospital mortality	754 (4.7)
Length of stay	9.16 ± 11.33 (0–109)

^a^Mean ± standard deviation (SD).

**Table 2:** Comorbidities and complications for corrosive injury patients, *N* = 16,001.

Variables	*N* (%)
Comorbidities	
Malignant neoplasms	**136 (0.8)**
Trachea, bronchus, and lung	38 (0.2)
Liver, primary	45 (0.3)
Hepatic flexure colon	24 (0.1)
Female breast	14 (0.1)
Upper lip, tongue, gum, mouth, palate, tonsil, hypopharynx, lip, oral cavity, and pharynx	15 (0.1)
Chronic disease	**1910 (11.9)**
Cerebrovascular disease	209 (1.3)
Diabetes	1062 (6.6)
Chronic lower respiratory disease	144 (0.9)
Liver cirrhosis	337 (2.1)
Chronic renal failure	339 (2.1)
Psychiatric disorder	**3687 (23.0)**
Depressive disorder	1952 (12.2)
Generalized anxiety disorder	188 (1.2)
Depression and anxiety	46 (0.3)
Other psychiatric disorders	1501 (9.4)
Complications	
Systemic complications	
Aspiration pneumonia	628 (3.9)
Respiratory failure	955 (6.0)
DIC	29 (0.2)
Septicemia	22 (0.1)
Acute renal failure	211 (1.3)
GI complications	
GI bleeding	234 (1.5)
GI perforation	69 (0.4)
Bleeding and perforation	6 (0.0)
Corrosive esophagitis	616 (3.8)
Peritonitis	156 (1.0)
Fistula	85 (0.5)
Esophageal stricture and stenosis	209 (1.3)

GI: gastrointestinal.

**Table 3:** Compare gender difference between male and female of corrosive injuries patients.

Variables	Male *N* (%)	Female *N* (%)	*p* value
Age			
0–18	699 (10.0)	544 (6.1)	<0.001
19–39	2739 (39.1)	3179 (35.4)	
40–64	2490 (35.5)	2986 (33.2)	
Above 65	1082 (15.4)	2282 (25.4)	
Suicide			
Yes	2246 (32.0)	3888 (43.2)	<0.001
No	4764 (68.0)	5103 (56.8)	
In-hospital mortality			
Yes	304 (4.3)	450 (5.0)	0.052
No	6706 (95.7)	8541 (95.0)	
Length of stay^a^	9.40 ± 11.60	8.97 ± 11.12	0.017
Comorbidities			
Psychiatric disorder			
Yes	1400 (20.0)	2287 (25.4)	<0.001
No	5610 (80.0)	6704 (74.6)	
Depressive disorders			
Yes	628 (9.0)	1370 (15.2)	<0.001
No	6382 (91.0)	7621 (84.8)	
Generalized anxiety disorder			
Yes	75 (1.1)	159 (1.8)	<0.001
No	6935 (98.9)	8832 (98.2)	
Depression and anxiety			
Yes	17 (0.2)	29 (0.3)	0.430
No	6993 (99.8)	8962 (99.7)	
Other psychiatric disorders			
Yes	714 (10.2)	787 (8.8)	0.002
No	6296 (89.8)	8204 (91.2)	
Systemic complications			
Yes	579 (8.3)	879 (9.8)	0.001
No	6431 (91.7)	8112 (90.2)	
GI complications			
Yes	242 (3.5)	346 (3.8)	0.201
No	6768 (96.5)	8645 (96.2)	

^a^Mean ± standard deviation (SD).

**Table 4:** Multivariate model of risk factor associated with in-hospital mortality in patients with corrosive injuries.

Variables	Odds ratio	95% confidence interval	*p*
Age			
0–18^*∗*^	—		
19–39	3.08	1.49–6.35	0.002
40–64	6.75	3.31–13.74	<0.001
Above 65	10.93	5.37–22.27	<0.001
Hospital characteristics			
Medical center^*∗*^	1.90	0.58–6.22	0.289
Regional hospital	1.01	0.30–3.41	0.992
Local hospital	—		
Clinics	0.00	0.00–0.00	1.000
Malignant neoplasms	2.23	1.37–3.62	0.001
Chronic disease	1.30	1.08–1.56	0.006
Psychiatric disorder	0.48	0.38–0.61	<0.001
Systemic complications	5.43	4.61–6.41	<0.001
GI complications	2.02	1.63–2.51	<0.001
Suicide	1.23	1.05–1.44	0.009

^*∗*^Reference group.
